# Evaluation of the Prognosis of COVID-19 Patients According to the Presence of Underlying Diseases and Drug Treatment

**DOI:** 10.3390/ijerph18105342

**Published:** 2021-05-17

**Authors:** Ejin Kim, Yong Chul Kim, Jae Yoon Park, Jiyun Jung, Jung Pyo Lee, Ho Kim

**Affiliations:** 1 Department of Public Health Sciences, Institute of Health and Environment and Graduate School of Public Health, Seoul National University, Seoul 08826, Korea; platin@snu.ac.kr; 2 Department of Internal Medicine, Seoul National University Hospital, Seoul 03080, Korea; imyongkim@gmail.com; 3 Department of Internal Medicine, Dongguk University Ilsan Hospital, Goyang-si 10326, Korea; nephrojyp@gmail.com; 4 Data management and Statistics Institute, Dongguk University Ilsan Hospital, Goyang-si 10326, Korea; bestjudy@hanmail.net; 5 Department of Internal Medicine, Seoul National University College of Medicine, Seoul 03080, Korea; 6 Kidney Research Institute, Seoul National University Hospital, Seoul 03080, Korea; 7 Department of Internal Medicine, Seoul National University Boramae Medical Center, Seoul 07061, Korea

**Keywords:** COVID-19, underlying disease, medical treatment

## Abstract

Certain underlying diseases such as diabetic mellitus and hypertension are a risk factor for the severity and mortality of coronavirus disease (COVID-19) patients. Furthermore, both angiotensin converting enzyme inhibitors (ACEi) and angiotensin II receptor blockers (ARBs) are controversial at role in the process of COVID-19 cases. The aim of the study was to investigate whether underlying diseases and taking ACEi/ARBs, affect the duration of hospitalization and mortality in patients with confirmed COVID-19. Medical usage claims data for the past three years until 15 May 2020, from the “CORONA-19 International Cooperation Research” project was used. We analyzed the medical insurance claims data for all 7590 coronavirus (COVID-19) patients confirmed by RT-PCR tests nationwide up to 15 May 2020. Among the comorbidities, a history of hypertension (hazard ratio [HR], 1.51; 95% confidence interval [CI], 1.056–2.158) and diabetes (HR, 1.867; 95% CI, 1.408–2.475) were associated significantly with mortality. Furthermore, heart failure (HR, 1.391; 95% CI, 1.027–1.884), chronic obstructive pulmonary disease (HR, 1.615; 95% CI, 1.185–2.202), chronic kidney disease (HR, 1.451; 95% CI, 1.018–2.069), mental disorder (HR, 1.61; 95% CI, 1.106–2.343), end stage renal disease (HR, 5.353; 95% CI, 2.185–13.12) were also associated significantly with mortality. The underlying disease has increased the risk of mortality in patients with COVID-19. Diabetes, hypertension, cancer, chronic kidney disease, heart failure, and mental disorders increased mortality. Controversial whether taking ACEi/ARBs would benefit COVID-19 patients, in our study, patients taking ACEi/ARBs had a higher risk of mortality.

## 1. Introduction

After its emergence in China in December 2019, the coronavirus disease (COVID-19) caused by the novel severe acute respiratory syndrome coronavirus 2 (SARS-CoV-2) spread to South Korea in January 2020. As of 18 April 2021, one year after the first outbreak of COVID-19 in Korea, the cumulative number of cases was 104,006, with a total of 1797 deaths (http://ncov.mohw.go.kr/, accessed on 18 April 2021), and the country was experiencing the fourth wave of the epidemic. The outbreak of the infection in specific groups, such as some religious groups, nursing homes, mental hospitals, and occupational groups (e.g., call centers and others) had epidemiological implications. The characteristics of these groups affected the prognosis of diseases according to their demographics and the presence or absence of underlying diseases [1,2,3]. Older age and the presence of underlying diseases have been shown to be factors leading to the severe stage of COVID-19 [4] and death of these patients [5,6]. In particular, the COVID-19 pandemic has shown that the presence of certain underlying diseases such as cancer, hypertension, stroke, and DM, are risk factors for severe disease and mortality of COVID-19 patients in various countries [7,8,9].

Furthermore, the angiotensin converting enzyme is used by SARS-CoV-2 to enter the target cell [10]. It has been reported that both angiotensin converting enzyme inhibitors (ACEi) and angiotensin II receptor blockers (ARBs) can significantly increase the mRNA expression of cardiac angiotensin-converting enzyme 2 [11]. Based on this evidence, it has been hypothesized that these drugs may play a role in COVID-19 disease severity [12]; however, there is a lack of evidence on the potential negative effects of these drugs on COVID-19 [13]. ACEi/ARBs are renin-angiotensin-aldosterone system (RAS) inhibiting agents and is commonly used anti-hypertensive agents to protect cardiovascular and cerebrovascular diseases, also in patients with chronic kidney disease for anti-proteinuric effects. Therefore, this concern remains to be elucidated.

We explored the risk factors related to the mortality and hospitalization of COVID-19 patients, and investigated their impact. In addition, we investigated the effects of underlying diseases (such as chronic kidney disease, hypertension, diabetes mellitus, ischemic heart disease, heart failure, cancer, chronic obstructive pulmonary disease, mental disorder, and cerebrovascular disease [14,15,16,17,18]) and a history of ACEi/ARBs treatment on mortality and length of hospital stay in these patients.

## 2. Materials and Methods

### 2.1. Patients and Data

Medical usage claims data for the past three years until 15 May 2020 from the “CORONA-19 International Cooperation Research” project hosted by the Ministry of Health and Welfare of the Republic of Korea and Health Insurance Intensive Assessment Service were used. In the Republic of Korea, under the entire national health insurance system, claims data that include all the costs incurred when using medical services at medical institutions nationwide, are routinely collected. This study used the national health insurance system claims database from three years prior to the date each patient was diagnosed with COVID-19 to 23 May 2020. Patients were defined as those diagnosed with COVID-19 based on by real-time reverse transcription polymerase chain reaction (RT-PCR) testing at the Korea Centers for Disease Control and Prevention by 15 May 2020. The mortality cases were included only during the COVID-19 infection status and it was excluded when patient died after COVID-19 infection was cleared up.

Using the specific International Classification of Diseases Tenth Revision codes, patients with the following underlying diseases for three years before the diagnosis of COVID-19 were selected: chronic kidney disease, hypertension, diabetes mellitus, ischemic heart disease, heart failure, cancer, chronic obstructive pulmonary disease, mental disorder, and cerebrovascular disease, as well as those with a history of ACEi/ARBs treatment. Patients who received an ACEi/ARBs prescription at least 2 times, and the next prescription interval after the first prescription was within 30 days were defined as those who received an ACEi/ARBs prescription by tracking the drug prescription history back from the patients claims history.

The Charlson Comorbidity Index (CCI) is the most widely used method among comorbidity index adjustment methods. It is a method to adjust the sum of these weights after assigning a constant weight from 1 to 6 points to 19 diseases defined through a medical records survey. In this study, we used the Quan algorithm [19] that combines the advantages of the existing algorithm. Additionally, the Quan algorithm is more code defined than other algorithms, which increases the chance to observe comorbid diseases. Therefore, it is thought that this study will be sensitively applied to define patients who have various diseases as underlying diseases at the same time [20] (Supplementary Table S1).

### 2.2. Statistical Analyses

Categorical variables were reported as frequencies (percentages with 95% confidence intervals (CIs)) and continuous variables as means (with standard deviations (SDs)) according to the type of distribution. We calculated Kaplan–Meier survival estimates and used the log-rank test to compare groups in terms of survival. The association of risk factors with time to death was assessed using univariable and multivariable Cox proportional hazards regression models. A generalized linear model was used to investigate the association of the risk factors with hospitalization. In order to investigate the factors affecting the length of hospital stay on of the prognostic indicators, a subgroup analysis was performed according to socioeconomic status that excluded patients who died during the study period.

Multivariable models were developed for demographics and diabetic mellitus and hypertension as the underlying disease comorbidities in the basic model, with drug treatment and additional underlying diseases added one by one, to measure their effect on mortality and hospitalization. In addition, the use of ACEi/ARBs, or history, and the use of a ventilation during the treatment were also applied to the model.

Cox proportional hazards regression was used to analyze whether the effect of the drug, which was discussed as a COVID-19 drug in the absence of a COVID-19 treatment, affects mortality, the prognosis of COVID-19 patients. The effect of the drug was investigated by adjusting for underlying diseases that increase the risk of death, and whether ACEi/ARBs was prescribed (Model 2), or whether the patient was treated with a ventilator (Model 3). In addition, to consider the importance of each underlying disease, we performed an analysis of the scores of the CCI. To reflect the level of management of the underlying disease, we conducted subgroup analysis for each group according to the disease severity; groups were also classified based on specific drug treatment. We used insurance types as surrogate variables to obtain economic information. To assess the degree of underlying disease management, the socioeconomic conditions that affect chronic disease management were divided into medical aid and National Health Insurance Service beneficiaries and then analyzed.

## 3. Results

### 3.1. Description of the Cohort

From a population of 234,427 close contacts with suspected SARS-CoV-2 infection from January 20 to May 15, 2020, 7590 patients were confirmed to be COVID-19 positive (mean ± SD, 46.65 ± 19.76; median, 48; range, 0.9–98; interquartile range [IQR], 27–73) and were analyzed. Table 1 shows the demographic clinical characteristics, comorbidities, and outcomes of these patients. Of these patients, 3095 (40.75%) were male and 4495 (59.22%) female. The mean length of hospital stay was 22.71 ± 13.94 days, and the mean length of intensive care unit (ICU) stay was 22.38 ± 13.92 days. Mental disorder was the most common comorbidity (2659 (35.03%)), followed by hypertension (1957 (25.78%)), chronic obstructive pulmonary disease (1812 (23.87%)), and diabetes mellitus (1635 (21.45%)).

### 3.2. Univariable Analysis

A 10-year increase in age was significantly associated with mortality (hazard ratio (HR), 2.76; 95% CI, 2.74–2.79; *p* < 0.001). Hypertension, heart disease, diabetes, malignant neoplasm, chronic obstructive pulmonary disease, chronic kidney disease, mental disorder, and a history of ACEi/ARBs treatment, before a COVID-19 diagnosis, were associated with increased mortality in the univariable analysis (Table 1).

**Figure 1:**
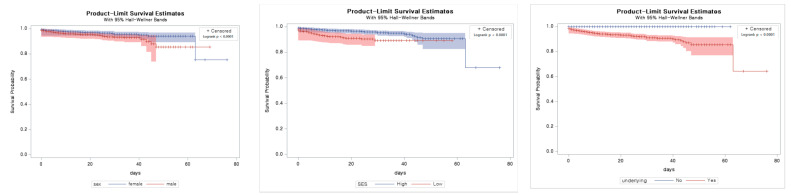
Kaplan–Meier Analysis of survival according to patients’ characteristics and conditions. SES socioeconomic status; underlying underlying disease.

We calculated Kaplan-Meier survival estimates and used log-rank tests to compare groups for each variable by sex, socioeconomic status, and underlying disease in terms of survival. It showed that there were differences in the estimated survival curve in the groups according to the sex, socioeconomic status, and underlying disease (Figure 1).

### 3.3. Observation Time and Main Outcomes

For the 7590 patients, the cumulative observation time was 164,329 patient-days from admission to the end of the follow-up (median observation time, (range, 1–128) days; IQR, 12–29 days) and there were 225 deaths (overall mortality, 2.96%). After a median follow-up of two days from admission to ICU transfer (IQR, 0–6; range, 0–47), there were 30 ICU deaths (13.3%). The distribution of the ICU length of stay is presented in Figure S1.

### 3.4. Multivariable Analysis

**Figure 2:**
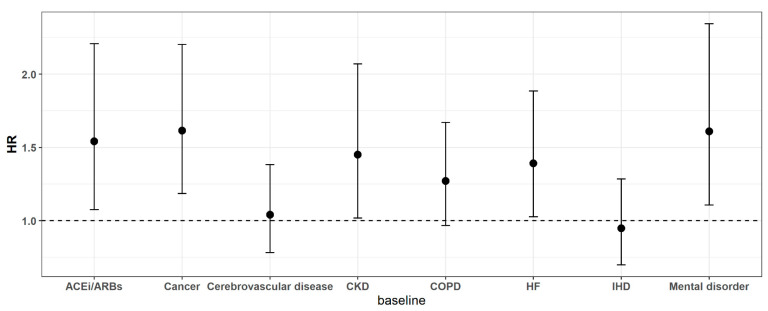
Effect of underlying conditions on mortality among COVID-19 patients. Note: ACEi/ARBs, cancer, cerebrovascular disease, CKD, COPD, HF, IHD, and mental disorder are added one by one to the model adjusting for the basic model gender, age, socioeconomic status, and underlying diseases such as diabetes and hypertension. ACEi/ARBs: angiotensin converting enzyme inhibitors (ACEi) and angiotensin II receptor blockers (ARBs); CKD: chronic kidney disease; COPD: chronic obstructive pulmonary disease; HF: heart failure; IHD: ischemic heart disease.

In the base model of the multivariable analysis, a year’s increase in age (HR, 1.105; 95% CI, 1.092–1.118) and male sex (HR, 2.475; 95% CI, 1.893–3.237) were significantly associated with mortality (Table S2). Among the comorbidities, a history of hypertension (HR, 1.51; 95% CI, 1.056–2.158) and diabetes (HR, 1.867; 95% CI, 1.408–2.475) were significantly associated with mortality. The results of the additional underlying diseases, heart failure (HR, 1.391; 95% CI, 1.027–1.884), cancer (HR, 1.615; 95% CI, 1.185–2.202), chronic kidney disease (HR, 1.451; 95% CI, 1.018–2.069), mental disorder (HR, 1.61; 95% CI, 1.106–2.343), and end stage renal disease (HR, 5.353; 95% CI, 2.185–13.12) showed a significant association with mortality. Regarding the association of a history of taking ACEi/ARBs with mortality, the HR was 1.541 (95% CI, 1.076–2.207) (Figure 2).

With respect to the effect of the drug regimen on the probability of death, the HR was 1.614 (95% CI; 1.208–2.157) for lopinavir/ritonavir (Kaletra), 17.62 (95% CI; 4.273–72.628) for rivabirin, 7.175 (95% CI; 5.401–9.530) for steroids, and 5.579 (95% CI; 3.435–9.062) for intravenous immunoglobulin (IVIG); these were statistically significant. On the other hand, the HR of hydroxychloroquine was 1.16 (95% CI; 0.879–1.529) (Table 2).

### 3.5. Subgroup Analyses

**Figure 3:**
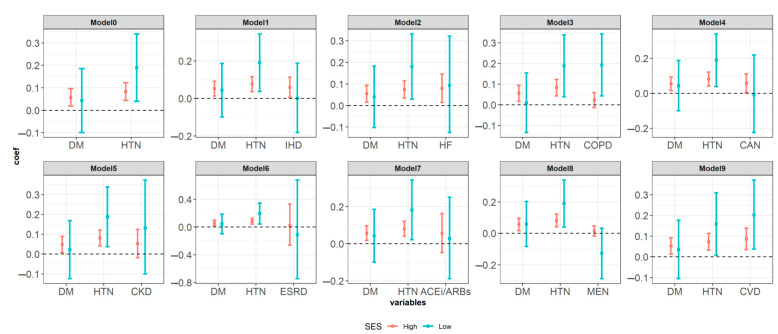
The effect of the underlying disease and whether or not taking ACEi/ARBs in the past or present on hospital stay according to the subgroup analysis based on the variable for classifying insurance types as a substitute variable for the socioeconomic status. Note: Model0: adjusted covariates age, sex and insurance type, Model1: adjusted covariates age, sex, insurance type and ischemic heart disease, Model2: adjusted covariates age, sex, insurance type and hear failure, Model3: adjusted covariates age, sex, insurance type and chronic obstructive pulmonary disease, Model4: adjusted covariates age, sex, insurance type and cancer, Model5: adjusted covariates age, sex, insurance type and chronic kidney disease, Model6: adjusted covariates age, sex, insurance type and end stage renal disease, Model7: adjusted covariates age, sex, insurance type and angiotensin converting enzyme inhibitors (ACEi) and angiotensin II receptor blockers (ARBs), Model8: adjusted covariates age, sex, insurance type and mental disorder. Model9: adjusted covariates age, sex, insurance type and cerebrovascular disease. DM, diabetic mellitus; HTN hypertension; IHD ischemic heart disease; HF heart failure; COPD chronic obstructive pulmonary disease; CAN cancer; CKD chronic kidney disease; ESRD end stage renal disease; ACEi/ARBs angiotensin converting enzyme inhibitors (ACEi) and angiotensin II receptor blockers (ARBs); MEN mental disorder; CVD cerebrovascular disease.

In the model based on age, sex, diabetes mellitus, and hypertension, in the group with high economic status, diabetes mellitus and hypertension had a statistically significant effect on the increase in hospital stay. In particular, hypertension was significant in all groups regardless of economic status. The size of the coefficient according to the economic status of hypertension was high in the group with low economic status. When each underlying disease was added to the basic model, hypertension (coefficient: 0.159, 95% CI: 0.008–0.310), cardiovascular disease (coefficient: 0.203, 95% CI: 0.038–0.372), a high socioeconomic status, and chronic obstructive pulmonary disease (coefficient: 0.1924, 95% CI: 0.0433–0.3431) were associated with a significantly longer duration of hospitalization (Figure 3).

**Figure 4:**
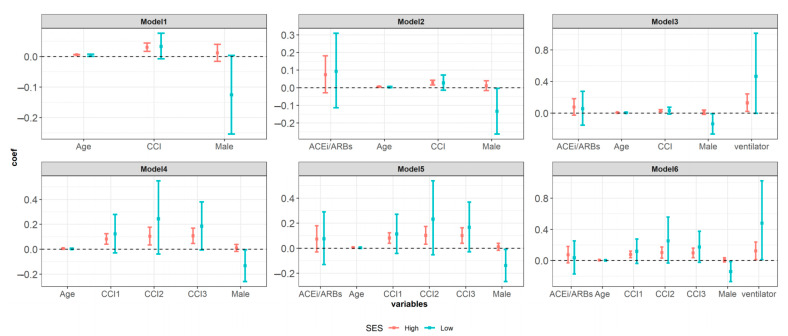
The effect of the Charlson Comorbidity Index and whether or not taking ACEi/ARBs in the past or present on hospital stay according to the subgroup analysis based on the variable for classifying insurance types as a substitute variable for the socioeconomic status. Note: Model1: adjusted covariates age, sex, insurance type and Charlson Comorbidity Index as continuous variables, Model2: adjusted covariates age, sex, insurance type, angiotensin converting enzyme inhibitors (ACEi) and angiotensin II receptor blockers (ARBs) and Charlson Comorbidity Index as continuous variables, Model3: adjusted covariates age, sex, insurance type, angiotensin converting enzyme inhibitors (ACEi) and angiotensin II receptor blockers (ARBs), ventilator and Charlson Comorbidity Index as continuous variables, Model4: adjusted covariates age, sex, insurance type and Charlson Comorbidity Index as categorical variables with reference CCI score0 (without underlying disease), Model5: adjusted covariates age, sex, insurance type, angiotensin converting enzyme inhibitors (ACEi) and angiotensin II receptor blockers (ARBs) and Charlson Comorbidity Index as categorical variables with reference CCI score0 (without underlying disease), Model6: adjusted covariates age, sex, insurance type, angiotensin converting enzyme inhibitors (ACEi) and angiotensin II receptor blockers (ARBs), ventilator and Charlson Comorbidity Index as categorical variables with reference CCI score is 0 (without underlying disease). CCI Charlson Comorbidity Index score; CCI1 Charlson Comorbidity Index score = 1; CCI2 Charlson Comorbidity Index score = 2; CCI3 Charlson Comorbidity Index score ≥ 3.

When the CCI was as a continuous variable, CCI were statistically significant at high socioeconomic status in Model 1 (coefficient: 0.0303, 95% CI: 0.0168–0.0441), Model 2 (coefficient: 0.029, 95% CI: 0.0154–0.0429 and Model 3 (coefficient: 0.0284, 95% CI: 0.0148–0.0423). When CCI was categorized by level, a higher CCI score was associated with a longer hospital stay. These results were statistically significant in patients with a high socioeconomic status. In patients with low socioeconomic status, the trend was similar, but was not statistically significant (Figure 4).

## 4. Discussion

The prediction of the prognosis of patients confirmed with COVID-19 is generally based on age and the presence or absence of an underlying disease. Men had a higher mortality rate than women, and older adults had a higher mortality rate than younger adults, which is consistent with the results of previous studies. [21,22,23,24]. In univariable analysis, the underlying disease had a significant effect on increasing the mortality rate [1,23,25]. To investigate the effect of patient mortality, not only sex and age but also diabetes, hypertension with high incidence rate and consistent management [25] and whether to take ACEi/ARBs [13], were applied as adjustment variables together to affect the COVID-19 fatality rate. In addition, cancer, chronic kidney disease, heart failure, and mental disorder were added to the model. Patients with these underlying diseases and patients with a history of ACEi/ARBs treatment showed a higher mortality rate. As a result of performing a subgroup analysis according to economic conditions, hypertension showed a significant effect on mortality at both economic levels, in all the models. This result was consistent with that of a previous study [26]. In a study conducted in Lombardy, Italy [27], inpatients receiving ICU care for COVID-19 had an increased risk of DM (HR, 1.18; 95% CI, 1.01–1.39), and in a study of patients hospitalized for COVID-19 in Spain [22], patients with cancer and DM had an increased mortality rate. In our study, cancer (HR: 1.615, 95% CI: 1.185–2.202) and DM (HR: 1.867, 95% CI: 1.408–2.475) in the base model and in the model that included other underlying diseases were associated with a significantly increased risk of mortality in patients with COVID-19 (Supplementary Table S2), which is consistent with the results of previous studies. Based on these results, there is a need to develop more elaborate treatment guidelines and strategies for managing common underlying diseases that are associated with increased mortality, in order to improve the prognosis of COVID-19 patients with underlying conditions.

Among COVID-19 patients, patients with an underlying disease had a higher mortality risk, and hypertension and CVD patients at all levels of socioeconomic status had longer hospital stays; however, patients with CVD as an underlying disease had longer hospital stays at a lower socioeconomic condition. Despite the government’s full support for the cost of COVID-19 treatment, these results suggest that the response to treatment of COVID-19 is affected by the degree of chronic disease control among vulnerable groups. Therefore, it is important to improve the management of chronic diseases that increase vulnerability to severe COVID-19. Due to the limitations in the billing data, the definitions of the ACEi/ARBs-prescribed patient group and operational definition of the level of economy were unclear. Despite the fact that the number of patients in the low-economic group occupied a small proportion of the total number of patients, it had a significant effect. In addition, data on clinical test results at an individual patient level were not available, so it was not possible to adjust for individual variations in response to treatment. However, despite these limitations, with the inclusion of all confirmed cases of COVID-19 in Korea, evaluating effects of the underlying diseases and drugs on the prognosis, provides evidence that could contribute to the prevention and treatment of COVID-19. In our study, the hospitalization of patients with low economic status, that is, patients with medical aid, was higher than those who were National Health Insurance Service beneficiaries. Unlike previous research findings that the type of insurance did not significantly affect the mortality rate, [28], in our study, when investigating the length of stay for alleviating diseases, excluding the deceased, the type of insurance had an effect on the length of hospital stay. This suggested that the management of the underlying disease may have had an effect on recovery; however, depending on the underlying disease, this effect was significant in both insurance types.

Our data showed that most of the deaths occurred within seven days of the average hospital stay; thus, the duration of hospitalization was an outcome, and when the subgroup analysis was performed by economic level, the effect of the underlying diseases was investigated after excluding deceased patients. In the low-economic group, even if the patient with underlying disease received the same quality of management as the patient in the high-economic group, the effect of the underlying disease may have indirectly influenced the patient’s prognosis.

For the same underlying disease, the low-economic group had a greater effect on hospital stay than the high-level group with hypertension and cerebrovascular bleeding being significant, and in particular, the effect of hypertension was significantly higher in all the models. The underlying diseases affecting the length of stay were hypertension and cerebrovascular disease in all the groups, regardless of the economic level. In the high-economic group, patients with diabetes, cardiovascular diseases such as ischemic heart disease, and heart failure, as well as cancer showed an increased probability of an increased length of hospital stay. On the other hand, for chronic obstructive pulmonary disease, this effect was statistically significant in the low-economic group. Chronic obstructive pulmonary disease was not significant in the analysis of the association between the underlying disease and effect of mortality, which was different from the findings of previous studies [29]. However, during the recovery process, it was found that underlying diseases whose management was neglected affected the group. Those with underlying diseases such as hypertension and cerebrovascular disease were more likely to have a longer length of hospital stay regardless of their economic level. Based on the overall results, it appears that patients with hypertension, diabetes, and cerebrovascular disease had a longer recovery time, and those with chronic obstructive pulmonary disease had a longer recovery in a group with low economic status. There is a limit to the adjustment of individual clinical conditions during hospitalization due to the characteristics of the claim data, although the inclusion of all COVID-19 patients nationwide will be meaningful for infectious diseases that originate from a specific group.

The use of hydroxychloroquine, lopinavir/ritonavir (Kaletra), rivabirin, steroids, and IVIG for the treatment of COVID-19 patients were analyzed by gender, age, and socioeconomic status while adjusting the underlying disease model, except in the case of hydroxychloroquine. In this case, it was found that there was an increased mortality rate of patients who were treated with it. Its use can be interpreted as treatment for the purpose of symptom relief in patients with severe symptoms, without a treatment option. After adjusting for whether ACEi/ARBs were prescribed or not, the effect of medicine prescription on mortality showed the same pattern as the above result. When adjusting for ventilator use, the effect of drug prescription on death, except for steroids was not statistically significant. The results of increasing the risk of COVID-19 death with the use of ACEi/ARBs were inconsistent with the results of previous studies, which are still controversial [30]. There is sufficient scientific evidence that ACEi/ARBs upregulates the human ACE2 receptor which has been shown to be a viral entry of SARS-CoV-2 into cells [31,32,33]. Therefore, the use of ACEi/ARBs has been suggested to increase the risk of COVID-19 infection and adverse outcomes with COVID-19 infection which is concordant with our data. Further large-scale epidemiological studies should be elucidated.

## 5. Conclusions

Our data includes COVID-19 patients across the country. In this data, underlying diseases such as diabetes, hypertension, cancer, chronic kidney disease, heart failure, and mental disorders increased mortality. It is controversial whether taking ACEi/ARBs would benefit COVID-19 patients but in our study, patients taking ACEi/ARBs had a higher risk of mortality. The underlying diseases affecting hospital stay excluding the deceased were hypertension and cerebrovascular disease regardless of the type of insurance, and COPD in the medical aid group and diabetes and heart failure in the medical insurance group had an effect on increasing the length of stay. In addition, the higher the comorbid disease index, the longer the hospital stay.

## Figures and Tables

**Table 1:** Demographic and clinical characteristics, comorbidities, and outcomes of 7590 patients with COVID-19.

Variables	Category		N 7590	%	Death 225	%	HR	95%CI	*p*-Value
Sex	Male		3095	40.78	121	53.78	1.105	1.095–1.116	<0.0001
	Female		4495	59.22	104	46.22			
Age	Mean ± SD		46.65 ± 19.76				1.107	1.096–1.117	<0.0001
Age group	~59		5458	71.91	16	7.11			
	60~69		1116	14.7	32	14.22	9.956	5.463–18.144	<0.0001
	70~79		606	7.98	64	28.44	36.237	20.938–62.715	<0.0001
	80~99		343	4.52	93	41.33	99.050	58.244–168.446	<0.0001
	90~		67	0.88	20	8.89	85.127	44.053–164.498	<0.0001
Socioeconomic status	High		6961	91.71	184	81.78			
	Low		629	8.29	41	18.22	2.575	1.835–3.613	<0.0001
Baseline condition									
Underlying disease		No	3247	42.78	2	0.89			
		Yes	4343	57.22	223	99.11	86.88	21.60–349.53	<0.0001
Chronic kidney disease		No	184	7229	95.24	81.78			
		Yes	361	4.76	41	18.22	4.565	3.238–6.435	<0.0001
	ESRD	No	7570	99.74	220	97.78			
		Yes	20	0.26	5	2.22	12.11	4.975–29.46	
	KT	No	7584	99.92	224	99.56			
		Yes	6	0.08	1	0.44	5.68	0.80–40.55	<0.0831
Hypertension	No		5633	74.22	45	20.00			
	Yes		1957	25.78	180	80.00	11.56	8.33–16.03	<0.0001
Diabetic mellitus	No		5955	78.46	82	36.44			
	Yes		1635	21.54	143	63.56	6.51	4.96–8.55	<0.0001
Ischemic heart disease	No		6290	91.17	164	72.89			
	Yes		670	8.83	61	27.11	3.97	2.96–5.34	<0.0001
Heart failure	No		7180	94.60	158	70.22			
	Yes		410	5.40	67	29.78	7.71	5.78–10.27	<0.0001
Cancer	No		7021	92.5	171	76.00			
	Yes		569	7.5	54	24.00	4.06	2.96–5.51	<0.0001
COPD	No		5778	76.13	93	41.33			
	Yes		1812	23.87	132	58.67	4.56	3.49–5.95	<0.0001
Mental disorder	No		4391	64.97	41	18.22			
	Yes		2659	35.03	184	81.78	8.54	6.08–11.99	<0.0001
Cerebrovascular disease	No		6586	90.33	142	63.11			
	Yes		734	9.67	83	36.89	5.50	4.18–7.22	<0.0001
ACE inhibitor or ARBs	No		7395	97.43	185	82.22	0.12	0.08–0.16	<0.0001
	Yes		195	2.57	40	17.78			
CCI	Mean ± SD		0.505 ± 1.164				1.447	1.389–1.507	<0.0001
CCI group	0		5604	73.84	61	27.11			
	1		1173	15.45	62	27.56	4.91	3.447–6.996	<0.0001
	2		314	4.14	18	8.00	5.359	3.165–9.075	<0.0001
	3		499	6.57	84	37.33	16.805	12.074–23.389	<0.0001
Events of post covid-19 Dx									
ICU Ventilator *	No		7479	98.54	195	86.67			
	Yes		111	1.46	30	38.67	26.75	20.12–35.57	<0.0001
									
Cardiac arrest	No		7556	99.55	192	85.33			
	Yes		34	0.45	33	14.67	55.74	38.15–81.44	<0.0001
Pneumonia	No		5017	67.29	61	27.11			
	Yes		2483	32.71	164	72.89	5.49	4.08–29.46	<0.0001
Arrhythmia	No		7571	99.75	218	96.89			
	Yes		19	0.25	7	3.11	12.46	5.86–26.5	<0.0001
Hospitalization	Mean ± SD		22.71 ± 13.94						
Q1 (~13)			1705		105	46.67			
Q2 (14~20)			2036		49	21.78	0.22	0.15–0.31	<0.0001
Q3 (21~29)			1914		32	14.22	0.13	0.08–0.20	<0.0001
Q4 (>29)			1935		39	17.33	0.10	0.06–0.16	<0.0001
Length of ICU stay	Mean ± SD		22.38 ± 13.92						
Q1 (~9)			7469		181	80.44			
Q2 (10~22)			39		14	6.22	22.03	12.75–38.07	<0.0001
Q3 (23~30)			37		13	5.78	18.54	10.54–32.60	<0.0001
Q4 (>30)			45		17	7.56	14.58	8.79–24.19	<0.0001
Medicines									
No medicine			3973	52.35	25	11.11			
Medicine			3617	47.65	200	88.89	8.24	5.43–12.50	<0.0001
Hydroxychloroquine	No		5494	72.38	85	37.78			
	Yes		2096	27.62	140	62.22	3.88	2.96–5.08	<0.0001
Lopinavir/ritonavir (Kaletra)	No		4927	64.91	67	29.78			
	Yes		2663	35.09	158	70.22	4.33	3.25–5.77	<0.0001
Rivabirin	No		7586	99.95	223	99.11			
	Yes		4	0.05	2	0.89	53.33	13.10–217.16	<0.0001
Interferon	No		7527	99.17	197	87.56			
	Yes		63	0.83	28	12.44	20.07	13.49–29.87	<0.0001
Steroid	No		7200	94.86	91	40.44			
	Yes		390	5.14	134	59.56	31.05	23.73–40.65	<0.0001
Immunoglobulin (IVIG)	No		7560	99.6	205	91.11			
	Yes		30	0.4	20	8.89	31.53	19.89–49.98	<0.0001

ICU ventilator *: including all patients who have received intensive care unit and ventilator treatment.

**Table 2:** Effect of treatments on mortality for hospitalization.

Medicines		Hydroxychloroquine			Lopinavir/Ritonavir (Kaletra)			Rivabirin			Steroid			Immunoglobulin (IVIG)		
Variables	Category	HR	95	CI	HR	95	CI	HR	95	CI	HR	95	CI	HR	95	CI
**Model1**																
Age		1.101	1.087	1.114	1.1	1.087	1.114	1.102	1.088	1.115	1.084	1.070	1.098	1.098	1.085	1.112
Sex	Male	2.385	1.823	3.120	2.344	1.792	3.066	2.387	1.825	3.122	1.825	1.391	2.395	2.22	1.694	2.910
Socioeconomic status	Low	1.574	1.118	2.217	1.62	1.150	2.282	1.588	1.127	2.238	1.592	1.126	2.252	1.504	1.065	2.123
Hypertension	Yes	1.406	0.972	2.034	1.425	0.987	2.058	1.396	0.965	2.019	1.388	0.955	2.017	1.607	1.114	2.319
Diabetic mellitus	Yes	1.656	1.231	2.228	1.617	1.200	2.179	1.67	1.241	2.249	1.455	1.079	1.961	1.517	1.124	2.049
Ischemic heart disease	Yes	0.79	0.568	1.098	0.774	0.557	1.076	0.795	0.571	1.106	0.742	0.535	1.030	0.809	0.583	1.123
Heart failure	Yes	1.417	1.021	1.968	1.366	0.984	1.896	1.418	1.020	1.971	1.296	0.929	1.807	1.38	0.993	1.917
Chronic obstructive pulmonary disease	Yes	1.196	0.904	1.584	1.21	0.915	1.600	1.195	0.902	1.582	1.12	0.843	1.487	1.137	0.856	1.510
Cancer	Yes	1.539	1.125	2.106	1.532	1.121	2.096	1.543	1.128	2.112	1.675	1.221	2.298	1.677	1.223	2.299
Chronic kidney disease	Yes	1.377	0.963	1.969	1.327	0.926	1.901	1.404	0.982	2.009	1.236	0.863	1.772	1.332	0.929	1.910
medicine	Yes	1.16	0.879	1.529	1.614	1.208	2.157	17.62	4.273	72.628	7.175	5.401	9.530	5.579	3.435	9.062
**Model2: Model1 + ACEi/ARBs**																
Age		1.1	1.086	1.113	1.099	1.086	1.113	1.101	1.087	1.114	1.083	1.070	1.097	1.097	1.084	1.111
Sex	Male	2.345	1.792	3.068	2.293	1.752	3.000	2.346	1.793	3.069	1.801	1.372	2.363	2.178	1.662	2.855
Socioeconomic status	Low	1.562	1.109	2.200	1.622	1.151	2.286	1.577	1.119	2.222	1.577	1.115	2.229	1.481	1.048	2.091
Hypertension	Yes	1.354	0.933	1.966	1.364	0.941	1.977	1.337	0.920	1.943	1.332	0.912	1.944	1.53	1.056	2.216
Diabetic mellitus	Yes	1.618	1.200	2.180	1.568	1.161	2.118	1.628	1.207	2.195	1.429	1.059	1.928	1.469	1.086	1.988
Ischemic heart disease	Yes	0.775	0.557	1.078	0.760	0.547	1.057	0.779	0.559	1.085	0.735	0.530	1.017	0.789	0.568	1.097
Heart failure	Yes	1.365	0.980	1.901	1.308	0.939	1.822	1.365	0.980	1.903	1.251	0.895	1.748	1.317	0.945	1.835
Chronic obstructive pulmonary disease	Yes	1.195	0.903	1.582	1.206	0.912	1.595	1.192	0.901	1.578	1.125	0.848	1.493	1.132	0.853	1.504
Cancer	Yes	1.549	1.132	2.118	1.545	1.130	2.112	1.555	1.137	2.127	1.696	1.237	2.325	1.695	1.236	2.323
Chronic kidney disease	Yes	1.36	0.951	1.945	1.311	0.915	1.877	1.385	0.968	1.980	1.205	0.839	1.729	1.322	0.923	1.894
medicine	Yes	1.127	0.853	1.490	1.630	1.219	2.180	18.4	4.461	75.924	7.138	5.373	9.483	5.854	3.600	9.520
ACEi/ARBs	Yes	1.448	1.004	2.088	1.505	1.046	2.166	1.487	1.033	2.141	1.391	0.967	2.000	1.577	1.095	2.272
**Model3: Model2 + Ventilator**																
Age		1.105	1.091	1.120	1.104	1.089	1.118	1.104	1.090	1.118	1.093	1.078	1.107	1.104	1.090	1.118
sex	Male	2.226	1.695	2.925	2.211	1.683	2.906	2.235	1.701	2.937	1.898	1.443	2.496	2.239	1.701	2.947
Socioeconomic status	Low	1.965	1.385	2.788	1.989	1.400	2.824	1.967	1.386	2.791	1.89	1.329	2.688	1.961	1.383	2.783
Hypertension	Yes	1.362	0.944	1.966	1.344	0.930	1.941	1.343	0.930	1.940	1.319	0.911	1.910	1.35	0.934	1.951
Diabetic mellitus	Yes	1.343	0.988	1.824	1.332	0.980	1.811	1.346	0.990	1.829	1.278	0.943	1.733	1.348	0.990	1.835
Ischemic heart disease	Yes	0.932	0.668	1.298	0.927	0.666	1.292	0.932	0.669	1.299	0.854	0.613	1.191	0.931	0.668	1.298
Heart failure	Yes	1.072	0.759	1.513	1.091	0.774	1.538	1.1	0.780	1.552	1.129	0.799	1.596	1.091	0.774	1.538
Chronic obstructive pulmonary disease	Yes	1.068	0.804	1.418	1.06	0.799	1.407	1.059	0.798	1.406	1.043	0.785	1.387	1.063	0.800	1.411
Cancer	Yes	1.787	1.302	2.453	1.796	1.308	2.465	1.793	1.306	2.462	1.841	1.339	2.530	1.774	1.291	2.439
Chronic kidney disease	Yes	1.227	0.848	1.775	1.194	0.826	1.728	1.216	0.841	1.757	1.136	0.785	1.643	1.213	0.839	1.755
medicine	Yes	0.836	0.627	1.115	1.168	0.861	1.586	2.728	0.652	11.412	4.345	3.141	6.011	0.9	0.526	1.540
ACEi/ARBs	Yes	1.531	1.055	2.223	1.504	1.038	2.179	1.5	1.035	2.174	1.412	0.976	2.043	1.479	1.016	2.151
Ventilator	Yes	13.454	9.776	18.515	12.265	8.883	16.936	12.614	9.220	17.257	4.818	3.379	6.870	13.243	9.396	18.665

## Data Availability

Under the management and supervision of the Ministry of Health and Welfare and the Health Insurance Review and Assessment Service of South Korea, data cannot be available.

## Supplementary Materials

The following are available online at https://www.mdpi.com/article/10.3390/ijerph18105342/s1, Figure S1: Distribution of age, hospitalization and ICU length of patients with COVID-19, Table S1: ICD-10 coding algorithms, Table S2: Effect of underlying conditions on mortality among COVID-19 patients.
