# Supplementary material for: Evaluation of the Prognosis of COVID-19 Patients According to the Presence of Underlying Diseases and Drug Treatment
Source: Int J Environ Res Public Health. 2021 May 17;18(10):5342. doi: 10.3390/ijerph18105342 (PMC8157012; doi:10.3390/ijerph18105342)
Supplement: Supplementary file 1 [file ijerph-18-05342-s001.zip › ijerph-1183428-supplementary file_proof_0516.pdf]

Supplementary Information

For

Evaluation of the Prognosis of COVID-19 Patients According to the Presence of Underlying Diseases and Drug Treatment

Table S1. ICD-10 coding algorithms.

Table S2. Effect of underlying conditions on mortality among COVID-19 patients.

Figure S1. Distribution of age, hospitalization and ICU length of patients with COVID-19.

Table S1. ICD-10 coding algorithms.

Disease	Quan's codes
AIDS/HIV	B24.x
Cerebrovascular disease	G45.x, G46.x, H34.0, I60.x-I68.x
Congestive heart failure	I09.9 I11.0, I13.0, I13.2, I25.5, I42.0, I42.5-I42.9, I43.x, P29.0
Chronic pulmonary disease	I27.8, I27.9, J40.x, J67.x, J68.4, J70.1, J70.3
Dementia	F03.x F05.1, G30.x, G31.1
Diabetes without chronic complication	E10.0, E10.6, E10.8, E11.0, E11.6, E11.8, E12.0, E12.1, E12.6, E12.8, E12.9, E13.0, E13.6, E13.8, E14.0, E14.6, E14.8
Diabetes with chronic complication	E10.5, E10.7, E11.5, E11.7, E12.2-E12.5, E12.7, E13.5, E13.7, E14.5, E14.7
Hemiplegia or paraplegia	G04.1, G11.4, G80.1, G80.2, G82.3-G82.5, G83.0-G83.4, G83.9
Mild liver disease	B18.x, K70.0-K70.2, K70.9, K71.3-K71.5, K74.0-K74.2, K76.0, K76.2-K76.4, K76.8, K76.9, Z94.4
Moderate or severe liver disease	I85.0, I85.9, I86.4, I98.2, K70.4, K71.1, K76.5
Any malignancy, including leukemia and lymphoma	C43.x, C88.0-C88.2, C90.2, C94.4, C97.x
Metastatic solid tumor	C80.x
Myocardial infection	I21.x, I22.x
Peripheral vascular disease	I70.x, I73.1, I73.8, I77.1, I79.0, I79.2, K55.1, K55.8, K55.9
Peptic ulcer disease	K25.0-K25.3, K25.9, K26.0-K26.3, K26.9, K27.0-K27.3, K27.9, K28.0-K28.3, K28.9
Rheumatologic disease	M06.0-M06.4, M06.8, M06.9, M31.5, M33.0, M33.1, M33.9, M35.1, M36.0
Renal disease	I12.0, I13.1, N03.2-N03.7, N05.2-N05.7, N19.x, N25.0, Z49.0-Z49.2, Z94.0, Z99.2

Table S2. Effect of underlying conditions on mortality among COVID-19 patients.

	base model			IHD			HF			COPD			Cancer			CKD			Mental disorder			Cerebrovascular disease			ESRD			ACEi/ARBs		
	HR	95 %CI		HR	95 CI		HR	95%CI		HR	95%CI		HR	95%CI		HR	95%CI		HR	95%CI		HR	95% CI		HR	95% CI		HR	95%CI	
Age	1.105	1.092	1.118	1.105	1.092	1.118	1.102	1.089	1.115	1.103	1.090	1.116	1.105	1.092	1.118	1.105	1.092	1.118	1.098	1.085	1.112	1.105	1.092	1.118	1.106	1.093	1.119	1.104	1.091	1.116
Sex (male)	2.475	1.893	3.237	2.478	1.895	3.241	2.43	1.858	3.178	2.447	1.872	3.200	2.45	1.873	3.205	2.451	1.874	3.206	2.514	1.924	3.287	2.469	1.887	3.231	2.529	1.932	3.312	2.406	1.839	3.149
SES (Low)	1.564	1.112	2.198	1.569	1.116	2.206	1.583	1.126	2.226	1.538	1.093	2.162	1.543	1.097	2.170	1.578	1.123	2.219	1.452	1.029	2.050	1.554	1.102	2.191	1.583	1.126	2.226	1.548	1.101	2.176
HTN (Yes)	1.51	1.056	2.158	1.525	1.063	2.188	1.425	0.990	2.052	1.522	1.064	2.176	1.468	1.025	2.102	1.474	1.030	2.110	1.454	1.016	2.082	1.501	1.047	2.151	1.478	1.034	2.114	1.423	0.989	2.047
DM (Yes)	1.867	1.408	2.475	1.875	1.413	2.488	1.842	1.388	2.444	1.807	1.359	2.402	1.8	1.356	2.391	1.741	1.300	2.332	1.796	1.354	2.383	1.861	1.403	2.469	1.834	1.382	2.433	1.802	1.356	2.396
				0.947	0.699	1.284	1.391	1.027	1.884	1.271	0.967	1.670	1.615	1.185	2.202	1.451	1.018	2.069	1.61	1.106	2.343	1.04	0.782	1.382	5.353	2.185	13.116	1.541	1.076	2.207

DM, diabetic mellitus ; HTN hypertension; IHD ischemic heart disease; HF heart failure; COPD chronic obstructive pulmonary disease; CAN cancer; CKD chronic kidney disease; ESRD end stage renal disease; ACEi/ARBs angiotensin converting enzyme inhibitors (ACEi) and angiotensin II receptor blockers (ARBs)

Figure S1. Distribution of age, hospitalization and ICU length of patients with COVID-19.

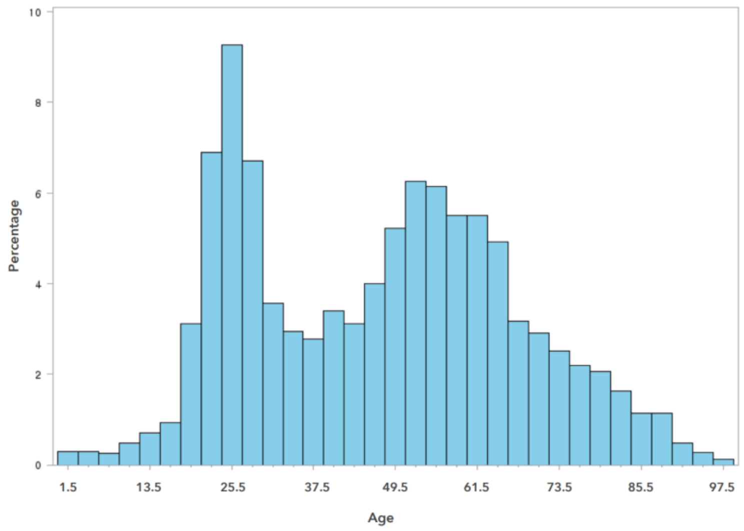

(a) Age

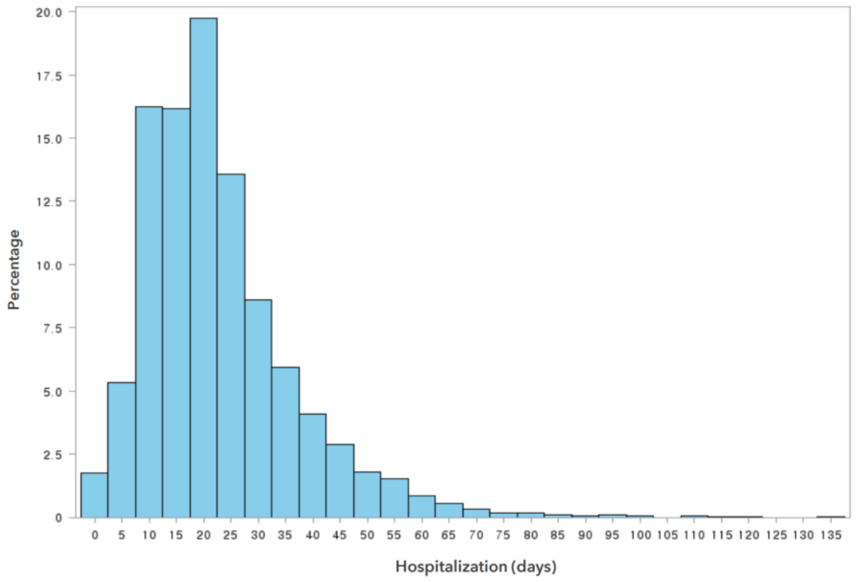

(b) Hospitalization

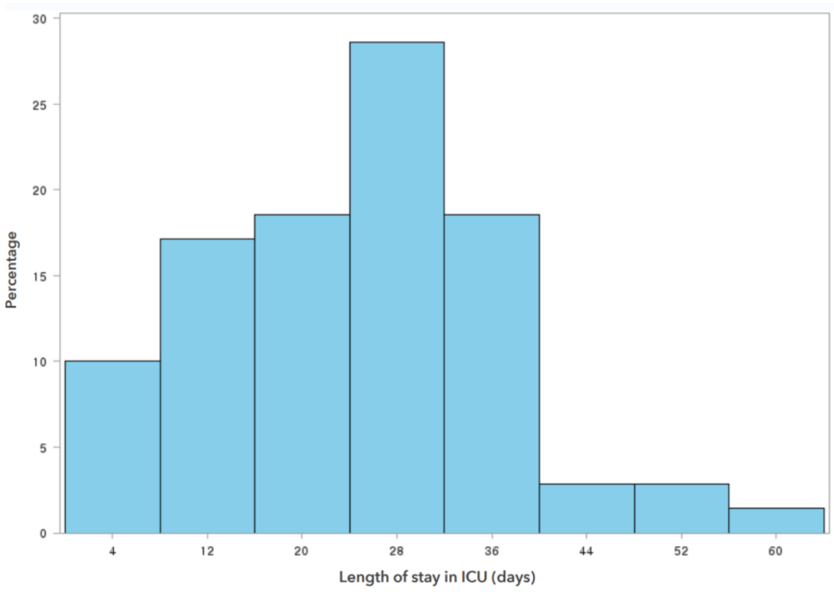

(c) Length of ICU stay

Figure S1. Distribution of age, hospitalization and ICU length of patients with COVID-19.
