# Supplementary figures and images for: Evaluation of the Prognosis of COVID-19 Patients According to the Presence of Underlying Diseases and Drug Treatment
Source: Int J Environ Res Public Health. 2021 May 17;18(10):5342. doi: 10.3390/ijerph18105342 (PMC8157012; doi:10.3390/ijerph18105342)

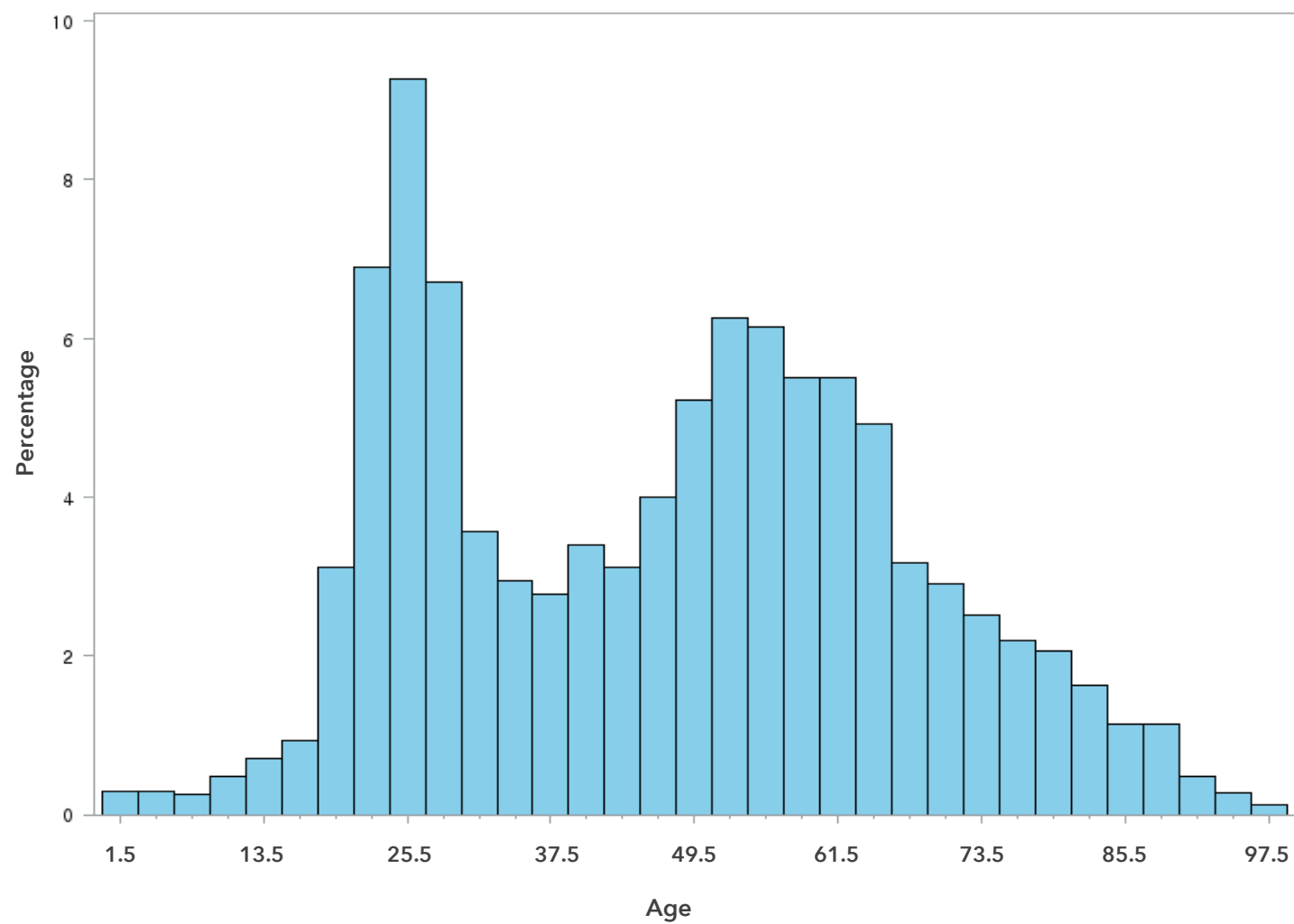

Supplement: Supplementary file 1 [file ijerph-18-05342-s001.zip › Supp_fig1_age.pdf]

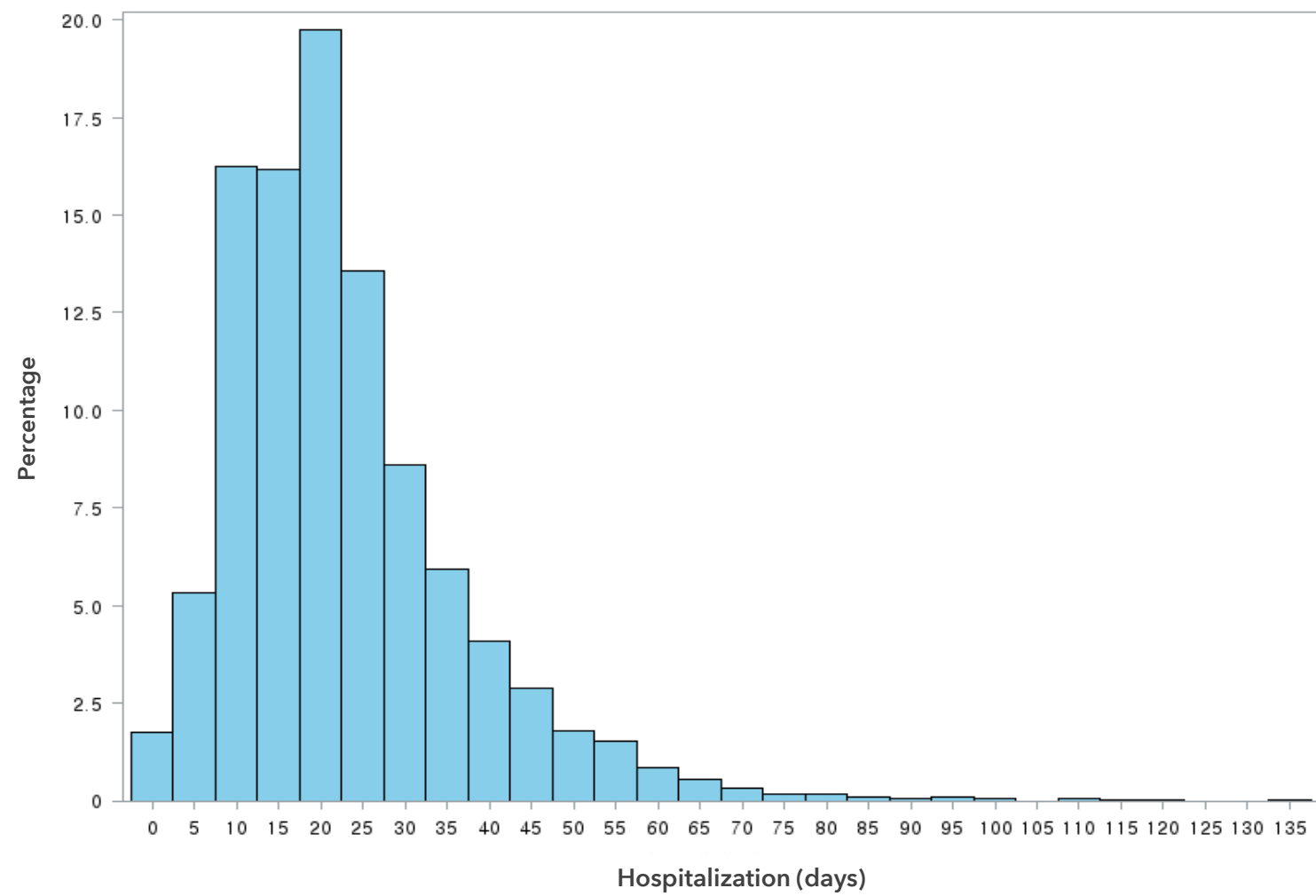

Supplement: Supplementary file 1 [file ijerph-18-05342-s001.zip › Supp_fig1_hospitalization.pdf]

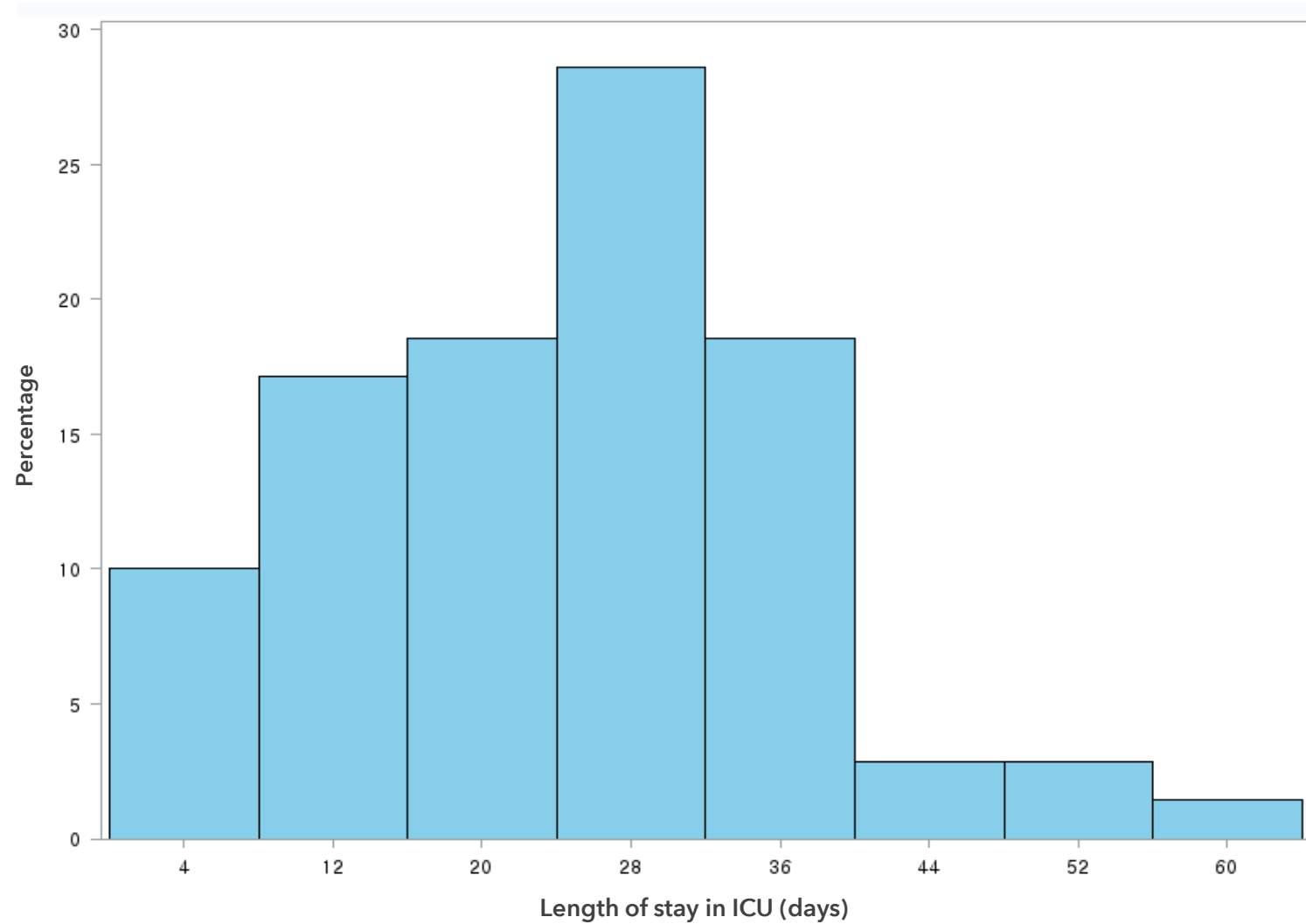

Supplement: Supplementary file 1 [file ijerph-18-05342-s001.zip › Supp_fig1_ICU.pdf]
